# Polydatin Restores Endothelium-Dependent Relaxation in Rat Aorta Rings Impaired by High Glucose: A Novel Insight into the PPARβ-NO Signaling Pathway

**DOI:** 10.1371/journal.pone.0126249

**Published:** 2015-05-05

**Authors:** Yang Wu, Lai Xue, Weimin Du, Bo Huang, Cuiping Tang, Changqing Liu, Hongmei Qiu, Qingsong Jiang

**Affiliations:** 1 Department of Pharmacology, Chongqing Key Laboratory of Biochemistry and Molecular Pharmacology, Chongqing Medical University, Chongqing 400016, PR China; 2 Pharmaceutical college, Fujiang medical University, Fujian 350004, PR China; Max-Delbrück Center for Molecular Medicine (MDC), GERMANY

## Abstract

Polydatin, a natural component from *Polygonum Cuspidatum*, has important therapeutic effects on metabolic syndrome. A novel therapeutic strategy using polydatin to improve vascular function has recently been proposed to treat diabetes-related cardiovascular complications. However, the biological role and molecular basis of polydatin’s action on vascular endothelial cells (VECs)-mediated vasodilatation under diabetes-related hyperglycemia condition remain elusive. The present study aimed to assess the contribution of polydatin in restoring endothelium-dependent relaxation and to determine the details of its underlying mechanism. By measuring endothelium-dependent relaxation, we found that acetylcholine-induced vasodilation was impaired by elevated glucose (55 mmol/L); however, polydatin (1, 3, 10 μmol/L) could restore the relaxation in a dose-dependent manner. Polydatin could also improve the histological damage to endothelial cells in the thoracic aorta. Polydatin’s effects were mediated via promoting the expression of endothelial NO synthase (eNOS), enhancing eNOS activity and decreasing the inducible NOS (iNOS) level, finally resulting in a beneficial increase in NO release, which probably, at least in part, through activation of the PPARβ signaling pathway. The results provided a novel insight into polydatin action, via PPARβ-NO signaling pathways, in restoring endothelial function in high glucose conditions. The results also indicated the potential utility of polydatin to treat diabetes related cardiovascular diseases.

## Introduction

Hyperglycemia is recognized as an independent and major risk factor that plays a causative role in development of diabetes-related cardiovascular complications, including microvascular diseases, such as neuropathy, retinopathy and nephropathy; as well as macrovascular diseases, such as stroke, coronary heart disease and peripheral artery disease [1–2]. Endothelial dysfunction caused by hyperglycemia is the common pathological basis of vascular disorders in diabetes mellitus (DM). The initial event of endothelial dysfunction is usually impaired endothelium-dependent relaxation (EDR), exhibited by attenuated vascular response to acetylcholine. Normally, vascular endothelial cells (VECs) regulate the function of local vascular smooth muscle cells (VSMCs) by maintaining a balance of endothelium-derived relaxing factor (EDRF, also known as nitric oxide, NO), prostaglandins (PGs), and enzymes that activate or degrade vasoactive hormones. In the case of DM, excessive cytokines, inflammatory factors and oxidative stress responses potentially alter the expressions and activities of endothelial NO synthase (eNOS) and cyclooxygenase-2 (COX_2_), which decrease the bioavailability of NO and prostacyclin (PGI_2_), thereby appearing to be responsible for EDR damage. Thus, besides lowering blood glucose, ways of improving the eNOS-NO or COX_2_-PGI_2_ system may provide significant protection against endothelial dysfunction and alleviate the vascular complications of DM.

Polydatin (3, 4', 5-trihydroxystibene-3-β-mono-*D*-glucoside) is one of the major active components extracted from *Polygonum Cuspidatum*, a traditional Chinese herbal medicine. It is also detected in red wine, grape hop cones, peanuts, hop pellets, chocolate products, cocoa-containing products and many daily diets. Substitution of the hydroxyl by a glycoside group in the C-3 position of resveratrol provides polydatin, with individual biological properties such as efficient absorption, resistance to enzymatic oxidation, and solubility in hot water [3–4]. Previously, pharmacological studies and clinical practice demonstrated that polydatin has important therapeutic effects in patients with cancer, hypertension, hyperlipemia, atherosclerosis, heart failure, diabetes and obesity [5–7]. Supplementation with polydatin could ameliorate experimental diabetes-induced fibronectin, reverse insulin resistance, and improve glucose and lipid metabolism [8–9]. In addition, in physiological concentrations with moderate red wine consumption, polydatin potentially increased the expression of eNOS and release of NO [10–11]. Thus, polydatin may possess a protective effect on endothelial cells in DM.

Recently, some nuanced interpretations have been proposed to explain certain observations about the beneficial effects of polydatin resulting from interactions with peroxisome proliferator-activated receptor transcription factors (PPARs), which comprise three isoforms, including α, β, γ. Extensive research has revealed the biological and pathophysiological roles of PPAR α and γ, which are promising pharmacological targets for type 2 diabetes and dyslipidemia, respectively. However, relatively little research has been performed on PPARβ, the only subtype of PPARs that is not the target of current drugs. However, genetically modified mouse models and newly developed synthetic ligands for PPARβ have demonstrated that PPARβ not only regulates genes involved in glucose and lipid metabolism, but also is involved in cardiovascular pathophysiology, making it a potential target for intervention of lifestyle-related diseases [12–13]. Highly selective PPARβ agonists GW501516, GW1516 and GW0742 improved endothelial function in diabetic mice [14]. Interestingly, our previous study found that polydatin could activate expression of PPARβ at both the mRNA and protein level in cardiomyocytes and diabetic mice (In press), which suggested that polydatin may be a PPARβ agonist and may also improve endothelial function. However, the effect of polydatin on EDR related to diabetes and the underlying mechanism of how polydatin triggers the signaling cascade remain largely unknown.

The present study aimed to observe the effect of polydatin on impaired EDR in related to DM by incubating isolated rat aortic rings in high glucose media. Meanwhile, different inhibitors were used to dissect the mechanism of its effects. This study will help to identify new targets for clinical therapy of diabetes-related cardiovascular diseases.

## Material and Methods

### Chemicals and reagents

Polydatin (C_20_H_22_O_8_; MW: 390.38; purity ≥ 95%) and GSK0660 were purchased from Sigma-Aldrich (St. Louis, MO, USA). Polydatin was dissolved in 1% dimethysulfoxide (DMSO) and directly diluted in medium to the required concentrations before the experiments. Acetylcholine, phenylephrine (PE), *N* ^*G*^-nitro-*L*-arginine methyl ester (*L*-NAME), meloxicam, horseradish peroxidase-conjugated goat anti-rabbit IgG and the nitrite detection kit were purchased from Beyotime (Jiang Su, China). RIPA lysis buffer, the BCA protein concentration assay kit (Enhanced), and the total NOS (tNOS) and eNOS detection kits were purchased from Nanjing Jiancheng Bioengineering Institute (Nanjing, China). Trizol and the reverse transcription kit were purchased from Takara BIO Inc (Otsu, Shiga, Japan). The anti-PPARβ, anti-eNOS, anti- inducible NOS (iNOS), anti-GADPH antibodies were purchased from Abcam (Cambridge, UK). All other reagents were of analytical grade. The gel imager and Quantity one software, the i-mark microplate reader and the icycler quantitative PCR instrument were purchased from BIO-RAD (Hercules, CA, USA).

### Preparation of rat thoracic aortas rings

All animal studies were performed in accordance with the Chongqing Management Approach Guide to the Care and Use of Laboratory Animals (Chongqing government order no. 195). All experiments involving rats were reviewed and approved by the Animal Laboratory Administration Center and Ethics Committee of Chongqing Medical University [SYXK (Chongqing) 2007–0001]. The vasoreactivity assay was based on the method of Qian *et al*., with some modifications [15]. Briefly, Sprague-Dawley rats (Animal Laboratory Center of Chongqing Medical University, Chongqing, China) (250–260 g; male and female) were anesthetized using 4% chloral hydrate (1 ml/100 g, *i*.*p*.) and then sacrificed by bleeding. The thoracic aorta was removed and cleaned of all loosely adherent tissues. Rings of aortas (3–4 mm long) were mounted in organ chambers filled with Krebs solution comprising (in mmol/L): NaCl 119, CaCl_2_ 2.5, KCl 4.7, MgSO_4_ 1.2, KH_2_PO_4_ 1.2, NaHCO_3_ 25.0, Glucose 11; pH 7.4. The Krebs was maintained at 37°C and with a continuous atmosphere of 95% O_2_/5% CO_2_.

### Measurement of the endothelium-dependent vasodilation

Vascular tone was recorded using a BL-420S Biological Signal Processing System provided by Chengdu Taimeng Science and Technology Co., Ltd. (Chengdu, Sichuan, China). The aortic rings were stretched to an optimal baseline tension. After equilibration for 1 h (replacing the solution every 15 min), the aortic rings were contracted by the application of PE (1 μmol/L). Once they reaching the steady state contraction, drugs were removed by several rinses with Krebs solution and the tension was allowed to return to baseline. The integrity of the endothelium was examined by testing the EDR of PE-precontracted rings under acetylcholine (10 μmol/L): a relaxation rate of 60% to 90% indicated an intact endothelium (77.01 ± 6.10% in our sets of experiments). Aortic rings were then incubated with Krebs solution containing normal glucose (NG, 11 mmol/L) or high glucose (HG, 55 mmol/L) for 6 h. Mannitol at 44 mmol/L was added in the NG control to adjust it to the equivalent osmolarity to HG. The aortic rings were rinsed with Krebs solution at least three times. The relaxant responses under acetylcholine (from 0.001 to 30 μmol/L with 0.5 logarithmic increments) were then studied in the PE pre-contracted rings. To study the effect of polydatin on EDR in hyperglycemic conditions, aortas were incubated with polydatin at different concentration (1, 3, or 10 μmol/L). In other experiments, in addition to polydatin (3 μmol/L), GSK0660 (1 μmol/L, a PPARβ blocker), *L*-NAME (10 μmol/L, an eNOS inhibitor), or meloxicam (100 μmol/L, a COX_2_ inhibitor) were added to investigate the possible mechanisms of polydatin. All experiments were repeated six times.

### Histological observation

For hematoxylin and eosin (HE) staining, thoracic aortas were fixed with 4% paraformaldehyde in a centrifuge tube after incubation for 6 h. The HE-stained aortas were then examined under light microscopy to assess the aortic rings. For transmission electron microscope observation, thoracic aortas were fixed with 2.5% cold glutaraldehyde solution (pH 7.4) in a centrifuge tube. They were rinsed and postfixed with 1% osmium tetroxide in 0.1 mol/L PBS for 2 h at room temperature, and then dehydrated through a graded series of ethanol to propylene oxide, and embedded in epoxy resin. 600-Å sections were made and observed under the transmission electron microscope.

### Quantitative real time RT-PCR (qRT-PCR) analysis of mRNA

Total RNA was extracted from aortic homogenates using the Trizol reagent, quantified by ultraviolet spectrometric detection (Eppendorf, Germany) and reverse transcribed into cDNA using a PrimeScript RT reagent kit (Takara Biotech Co., Dalian, China), according to the manufacturer’s instructions. QRT-PCR was performed to analyze mRNA expression of *PPARβ*, *eNOS* and *iNOS*, according to the standard protocol of the SYBR *Premix Ex Taq* II kit (Takara Biotech Co., Dalian, China). The primers used for SYBR green qRT-PCR were synthesized by Takara Biotech Co. (see Table 1). The amount of target gene mRNA relative to the internal control gene, GADPH, was calculated using the ΔCt (Ct = cycle threshold) method as follows: the relative expression = 2‾^ΔCt^, ΔCt = Ct (target gene)−Ct (GADPH). The results of three independent experiments were used for statistical analysis.

**Table 1:** PCR primers for gene amplification.

Gene	Forward (5′-3′ orientation)	Reverse (5′-3′ orientation)
*PPARβ*	CTGGCAGAACCCAGTACCAG	GTGAGCCGGTGTCATGGTTA
*eNOS*	TCACCGATACAACATACTTG	TCAGAGCCATACAGGATAGT
*iNOS*	TGGTGAGGGGACTGGACTTT	CCAACTCTGCTGTTCTCCGT
*GADPH*	CCATCACCATCTTCCAGGAG	CCTGCTTCACCACCTTCTTG

### Western blotting analysis of proteins

Isolated protein samples from aortic homogenates were separated by sodium dodecyl sulfate—polyacrylamide gel electrophoresis. The proteins (70 μg) were then transferred to polyvinylidene difluoride membranes and incubated with primary monoclonal mouse anti-PPARβ (1:700 dilution), anti-eNOS (1:1000 dilution) and anti-iNOS (1:1000 dilution) antibodies overnight, and then with the corresponding peroxidase-conjugated secondary antibodies (1:1000 dilution) on the second day. Antibody binding was detected using an ECL detection kit (Amersham Biosciences, Piscataway, NJ, USA) and densitometric analysis was performed using a quantitative imaging system (Bio-Rad, USA). All western blotting experiments were repeated three times.

### Measurement of eNOS and iNOS activities

For NOS activity, the tNOS (constitutive NOS [cNOS] + inducible NOS [iNOS]) and the eNOS activity were assayed in accordance with the kit manual (in rat aortas the cNOS = eNOS). The iNOS activity was calculated by tNOS minus eNOS. The enzyme activities were expressed as units per mg of protein. The results of six independent experiments were used for statistical analysis.

### Measurement of NO and PGI_2_ release

Levels of the NO derivative nitrite were determined in the supernatant obtained from the aortic homogenate using the Griess reaction. A nitrite detection kit was used according to the manufacturer’s instructions, and a standard curve using NaNO_2_ was generated for quantification. Briefly, 50 μl of medium or standard NaNO_2_ was mixed with 50 μl of Griess Reagent I and 50 μl of Griess Reagent II in a 96-well plate. After 15 min, the optical density was read in a microplate reader (Tecan Austria Ges.m.b.H) at 540 nm. The PGI_2_ concentration was measured using a PGI_2_ ELISA kit, according to the manufacturer’s instructions. The optical density values of the samples were measured at 450 nm with a microplate reader. The concentration was calculated according to a standard curve. The results of six independent experiments were used for statistical analysis.

### Statistical analysis

Results were expressed as means ± S.E.M. Relaxation rates of respective concentrations in each group obtained in response to cumulative concentrations of acetylcholine were expressed as percent change in the level of tone induced by PE. E_max_ represented the maximal drug relaxation effect of acetylcholine in each group. The negative log molar concentration of acetylcholine for inducing 50% of E_max_, pD_2_, was calculated from the concentration-response curve by linear regression. Statistical differences were tested using one-way ANOVA. *P* values < 0.05 were considered statistically significant.

## Results

### Effect of polydatin on EDR induced by acetylcholine

**Fig 1:**
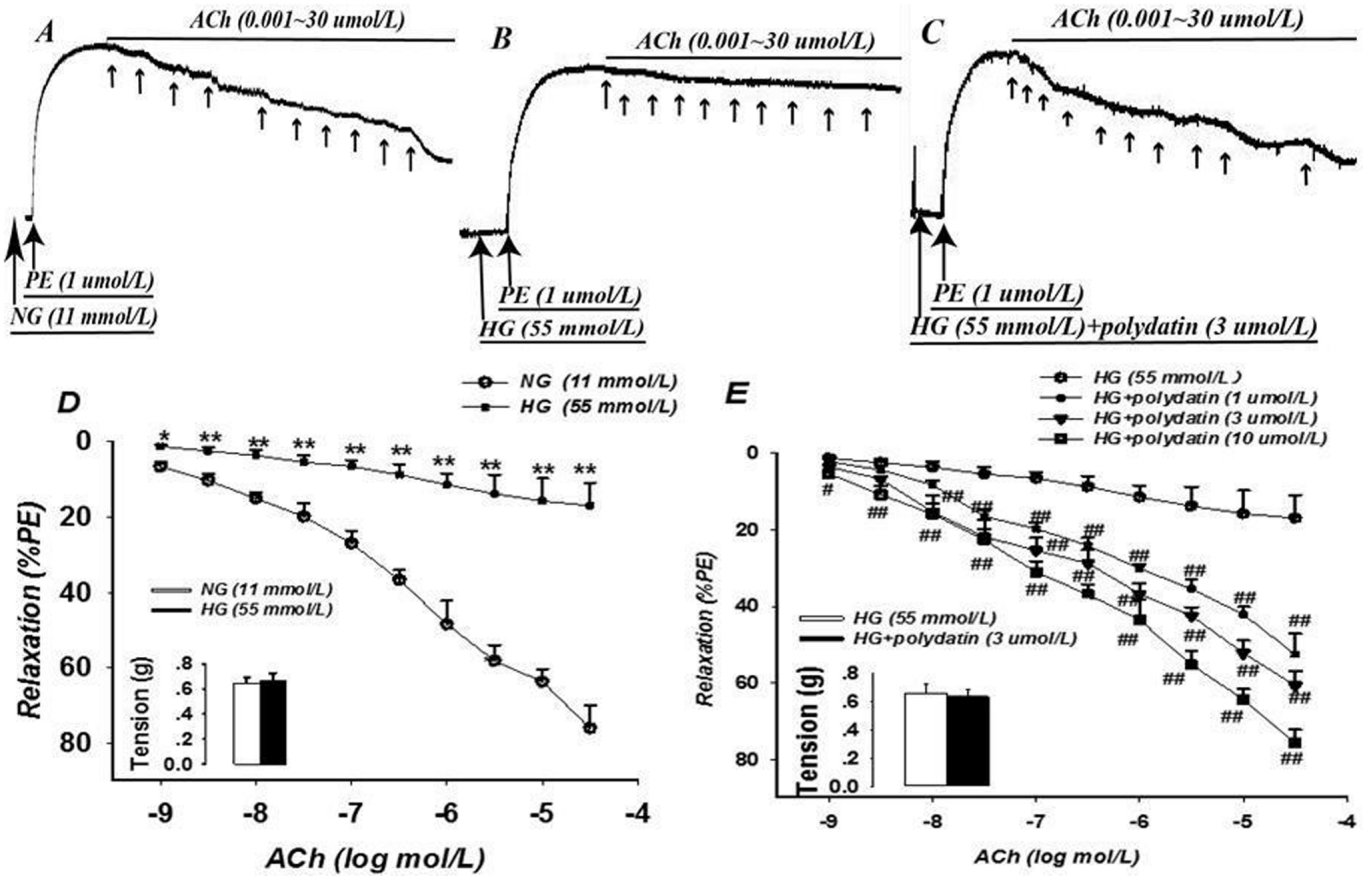
Effect of polydatin on endothelium-dependent relaxation of acetylcholine under HG (glucose at 55 mmol/L). The vascular relaxant responses induced by acetylcholine (ACh) from 0.001 to 30 μmol/L in arteries pre-contracted by phenylephrine (PE) at 1 μmol/L were significantly impaired by HG incubation. Co-incubation of polydatin (1, 3, or 10 μmol/L) in HG conditions for 6 h improved endothelium-dependent relaxation of acetylcholine, in a concentration-dependent manner. A-C: The typical traces of the dose-response relationship for ACh on rat aorta; D, E: The dose-response curve of ACh on aortic rings. Additionally, the bars of D and E indicate the contractility of PE-contracted aortic rings (mean ± S.E.M, n = 6). * *P*<0.05, ** *P*<0.01 *vs*. NG (glucose at 11 mmol/L); ^#^ *P*<0.05, ^##^ *P*<0.01 *vs*. HG.

After incubating for 6 h in HG, the arterial rings contracted with PE (1 μmol/L) showed similar contractility to NG (*P*>0.05), suggesting that high glucose had little influence on PE-induced vasoconstriction. Acetylcholine (from 0.001 to 30 μmol/L) caused a concentration-dependent relaxation under NG conditions (Fig 1A and 1D). This acetylcholine-induced relaxation was significantly decreased in HG-incubated samples (Fig 1B and 1D): the E_max_ and pD_2_ were reduced by 77.7% and 206.6%, respectively (*P*<0.05) (Table 2). Notably, the E_max_ was 16.96 ± 6.11% in the HG groups, which demonstrated impaired EDR. Co-incubation with polydatin (1, 3, or 10 μmol/L) markedly improved the impaired relaxation response to acetylcholine in HG, in a concentration-dependent manner (Fig 1C and 1E): the E_max_ increased by 2.1, 2.6, and 3.5 times, respectively; and the pD_2_ also significantly increased (*P*<0.05) (Table 2). Polydatin at 10 μmol/L could restore the relaxation under acetylcholine in HG to that of the NG control.

**Table 2:** Effect of polydatin on E_max_ and pD_2_ of acetylcholine in endothelium-dependent relaxation under HG (glucose at 55 mmol/L) conditions (mean ± S.E.M., n = 6).

Group	NG	HG	HG+Polydatin (1 μmol/L)	HG+Polydatin (3 μmol/L)	HG+Polydatin (10 μmol/L)
E_max_ (%)	76.10 ± 6.10	16.96 ±6.11^##^	52.56 ±5.44**	60.71 ±3.8**	75.78 ±3.53**
pD_2_	5.88 ± 0.11	-6.27 ±5.96^##^	4.31 ±0.29**	5.07 ±0.24**	5.85 ±0.13**

Note: E_max_: the maximal drug relaxation effect of acetylcholine; pD_2_: the negative log molar concentration of acetylcholine for inducing 50% of E_max_; NG: glucose at 11 mmol/L.

^##^ *P*<0.01 *vs* NG;

** *P*<0.01 *vs* HG.

### Effects of different inhibitors on polydatin on EDR under high glucose condition

**Fig 2:**
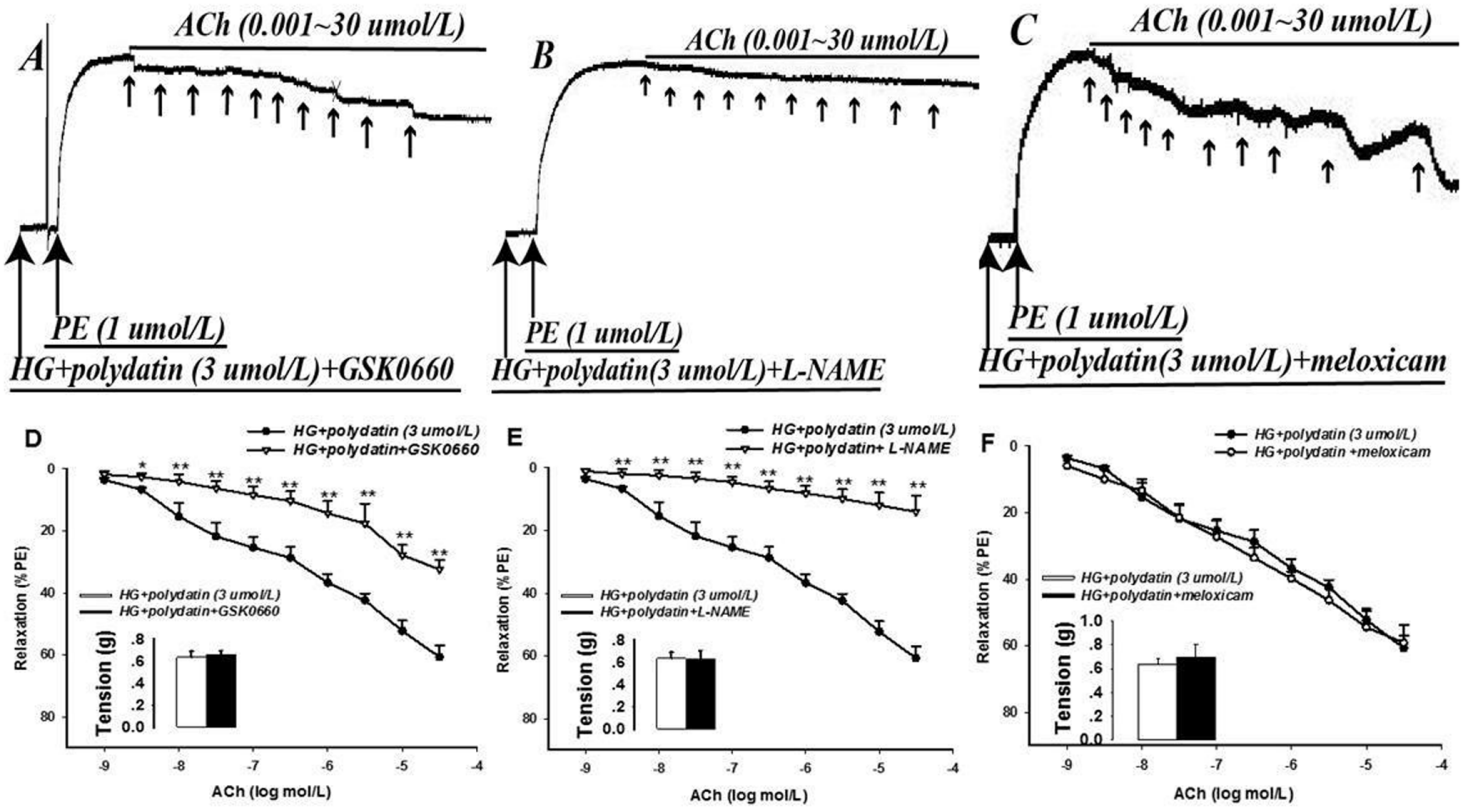
Effects of different inhibitors on polydatin on the impaired endothelium-dependent relaxation induced by HG (glucose at 55 mmol/L). Treatment with GSK0660 (1 μmol/L) could partially abolish the improved effect of polydatin (3 μmol/L) on the impaired relaxation of acetylcholine (ACh) induced by HG, the E_max_ and pD_2_ were significantly lower. *L*-NAME (10 μmol/L) could completely ablate the effect of polydatin. However, meloxicam (100 μmol/L) had no effect. A-C: The typical traces of the dose-response relationship for ACh on rat aorta; D-F: The dose-response curve of ACh on aortic rings. Additionally, the bars of D, E, and F indicate the contractility of phenylephrine (PE)-contracted arterial rings (mean ± S.E.M, n = 6). * *P*<0.05, ** *P*<0.01 *vs*. HG+polydatin (3 μmol/L).

Co-treatment with GSK0660 (1 μmol/L) partially abolished the improved effect of polydatin on the impaired relaxation under acetylcholine in HG (Fig 2A and 2D): the E_max_ and pD_2_ were significantly lowed by 46.6% and 81.1%, respectively (*P*<0.01) (Table 3). Notable *L*-NAME (10 μmol/L) could completely ablate the effect of polydatin (*P*<0.01) (Fig 2B and 2E): the E_max_ and pD_2_ were decreased by 76.9% and 285.8% (*P*<0.01), which was close to the level of the HG group (*P*>0.05) (Tables 2 and 3). However, treatment with meloxicam (100 μmol/L) had no effect (*P*>0.05) (Fig 2C and 2F; Table 3).

**Table 3:** Effect of polydatin with different inhibitors on E_max_ and pD_2_ of acetylcholine in endothelium-dependent relaxation under HG (glucose at 55 mmol/L) conditions (mean ± S.E.M., n = 6).

Group	Polydatin	Polydatin+GSK0660	Polydatin+*L*-NAME	Polydatin+Meloxicm
E_max_(%)	60.71 ±3.8	32.41 ±3.11**	14.04 ±5.2**	59.14 ±5.37
pD_2_	5.07 ±0.24	0.96 ±0.5**	-9.42 ±5.07**	5.21 ±0.19

Note: E_max_: the maximal drug relaxation effect of acetylcholine; pD_2_: the negative log molar concentration of acetylcholine for inducing 50% of E_max;_ *L*-NAME_:_ *N* ^*G*^-nitro-*L*-arginine methyl ester.

** *P*<0.01 *vs* Polydatin.

### Effect of polydatin on histological morphology of aortic rings under high glucose conditions

**Fig 3:**
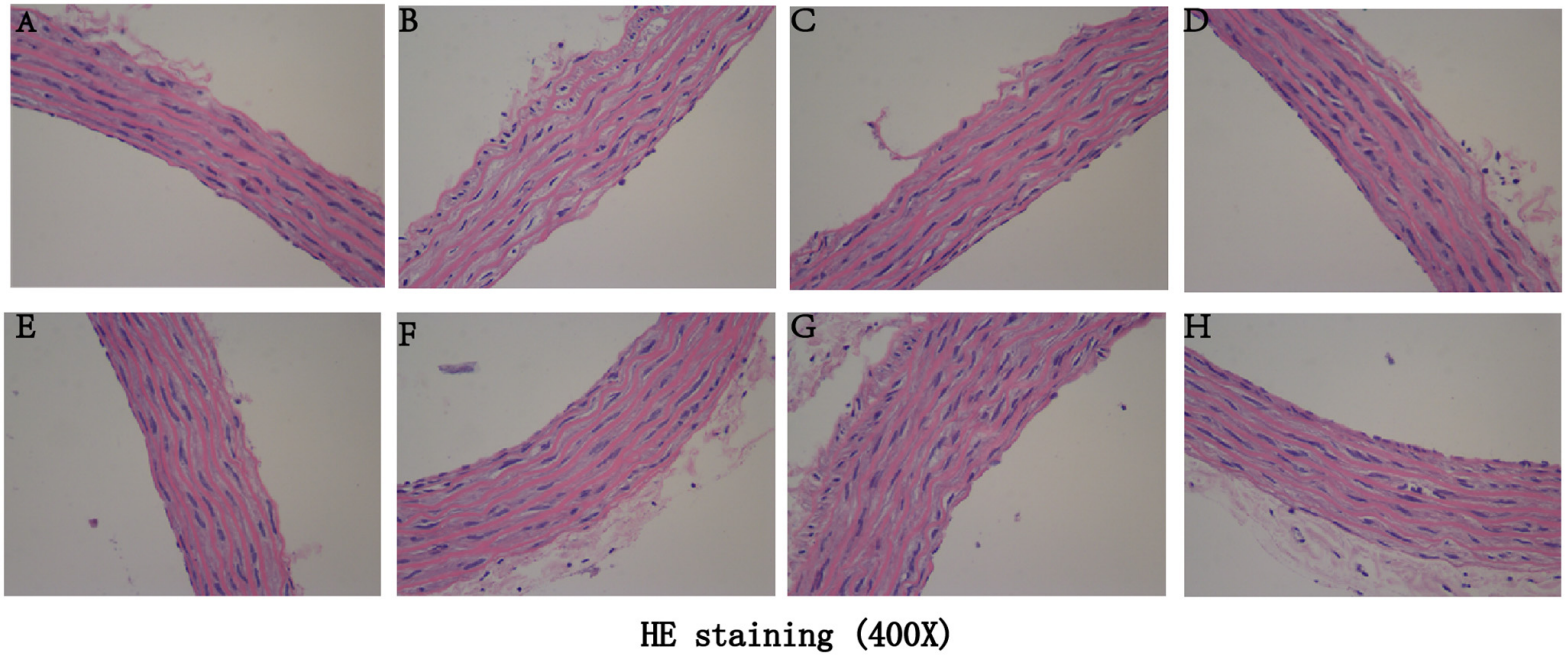
Effect of polydatin on morphological changes of aortic rings under high glucose conditions, as shown by hematoxylin and eosin staining (400×). (A) The control group of normal glucose (11 mmol/L) showed normal endothelium and aortic structure, which was significantly disturbed by high glucose (55 mmol/L) incubation (B). Treatment with polydatin (C: 1 μmol/L, D: 3 μmol/L, E: 10 μmol/L) inhibited the effect of high glucose (55 mmol/L) in a concentration-dependent manner. Notably, GSK0660 (F) or *L*-NAME (G), but not meloxicam (H), significantly reduced the effect of polydatin (3 μmol/L).

**Fig 4:**
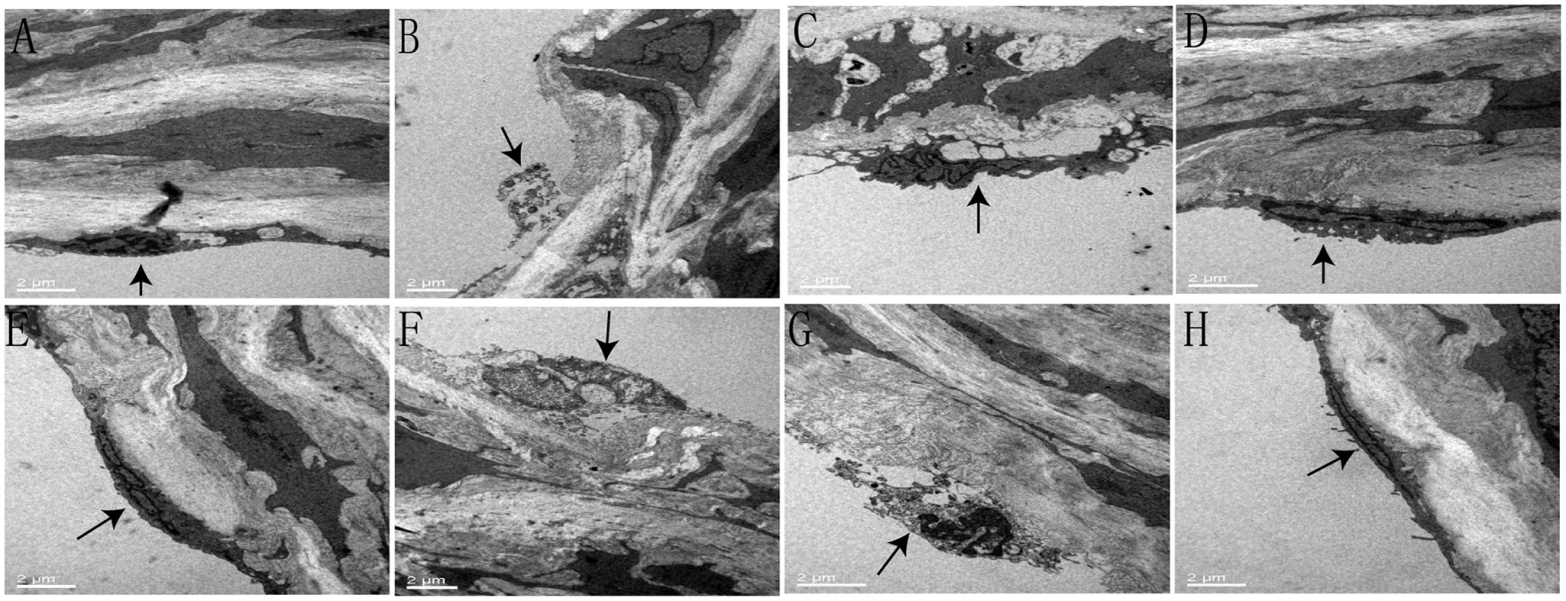
Effect of polydatin on morphological changes of aortic rings under high glucose conditions, as shown by transmission electron microscope (7000×). The control group of normal glucose (11 mmol/L) (A) showed normal endothelium, which was significantly disturbed by high glucose (55 mmol/L) incubation (B). Treatment with polydatin (C: 1 μmol/L, D: 3 μmol/L, E: 10 μmol/L) inhibited the effect of high glucose (55 mmol/L) in a concentration-dependent manner. Notably, GSK0660 (F) or *L*-NAME (G), but not meloxicam (H), significantly reduced the effect of polydatin (3 μmol/L).

As shown in Figs 3 and 4, in the NG group (Figs 3A and 4A), the intima of the thoracic aorta was smooth. The endothelial cells were flat and intact, and located closely to the internal elastic lamina. In addition, pinocytosis vesicles were abundant in the endothelial cytoplasm. However, after HG incubation (Figs 3B and 4B), the integrity and continuity of aortic intima were significantly disturbed: the endothelial cells were totally dissolved, the internal elastic lamina were fractured and partially dissolved. Moreover, the smooth muscle cell and their mitochondria were swollen. In the polydatin-treatment groups (Figs 3C–3E and 4C–4E), the impaired aortic intima caused by HG incubation were improved in a concentration-dependent manner (1, 3 and 10 μmol/L), the dissolved endothelial cells in the polydatin (10 μmol/L) group (Figs 3E and 4E) were similar to that of the NG group. Intriguingly, co-treatment with GSK0660 (1 μmol/L) (Figs 3F and 4F) or *L*-NAME (10 μmol/L) (Figs 3G and 4G) dramatically reduced the effect of polydatin (3 μmol/L). However, the inhibitor of COX_2_, meloxicam, had no influence to polydatin benefits (Figs 3H and 4H).

### Effect of polydatin on the mRNA and protein expression of PPARβ, eNOS and iNOS under high glucose conditions

**Fig 5:**
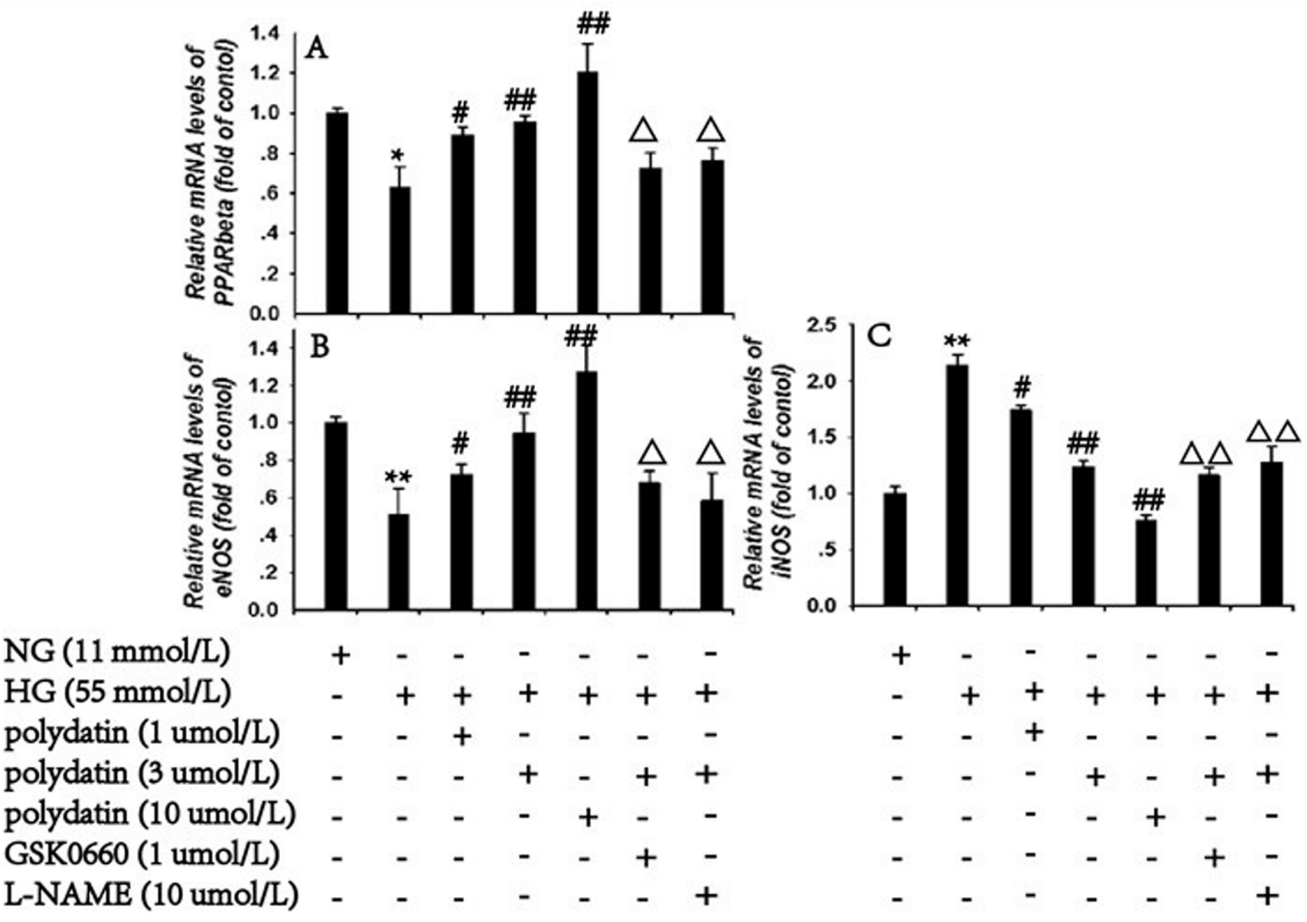
Effect of polydatin on the mRNA expression of PPARβ, eNOS and iNOS under HG (glucose at 55 mmol/L) conditions. Co-incubation with polydatin (1, 3, or 10 μmol/L) in HG-stimulated aortas markedly upregulated the decreased *PPARβ* and *eNOS* mRNA levels, and reduced the *iNOS* level in a dose-dependent manner. GSK0660 (1 μmol/L) or *L*-NAME (10 μmol/L) blocked the effects of polydatin (3 μmol/L). (mean ± S.E.M, n = 3). * *P*<0.05, ** *P*<0.01 *vs*. NG (glucose at 11 mmol/L). ^#^ *P*<0.05, ^##^ *P*<0.01 *vs*. HG. ^Δ^ *P*<0.05, ^ΔΔ^ *P*<0.01 *vs*. HG+polydatin (3 μmol/L). “+” or “-”: treatment with or without the relevant reagent.

**Fig 6:**
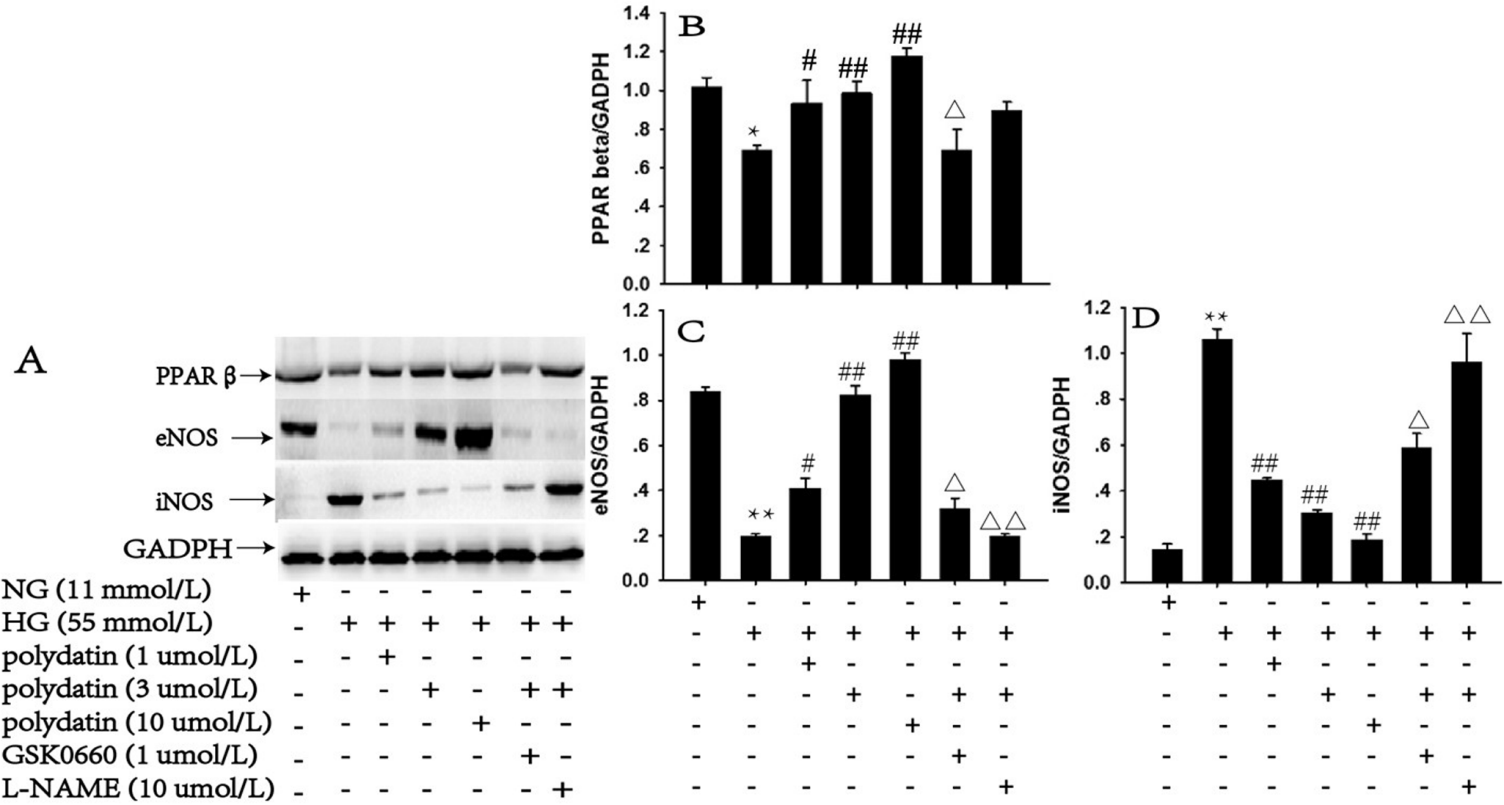
Effect of polydatin on the protein levels of PPARβ, eNOS and iNOS under HG (glucose at 55 mmol/L) conditions. Treatment with polydatin (1, 3, or 10 μmol/L) upregulated the decreased protein levels of PPARβ and eNOS, and reverse the increased level of iNOS induced by HG. The selective blocker of PPARβ, GSK0660, or eNOS antagonist *L*-NAME, clearly blocked the effects of polydatin (3 μmol/L). (mean ± S.E.M, n = 3). * *P*<0.05, ** *P*<0.01 *vs*. NG (glucose at 11 mmol/L). ^#^ *P*<0.05, ^##^ *P*<0.01 *vs*. HG. ^Δ^ *P*<0.05, ^ΔΔ^ *P*<0.01 *vs*. HG+polydatin (3 μmol/L). “+” or “-”: treatment with or without the relevant reagent.

HG incubation visibly downregulated both the mRNA and protein expression of PPARβ and eNOS, by 36.8% and 48.8%, and 32.12% and 76.72%, respectively. The mRNA and protein expressions of iNOS were increased by 114.3% and 6.4 times, compared with the NG group (*P*<0.05). Polydatin (1, 3, or 10 μmol/L) significantly elevated the PPARβ and eNOS expression levels, and decreased iNOS in a concentration-dependent manner (*P*<0.05). The effects of polydatin (3 μmol/L) on PPARβ, eNOS and iNOS mRNA and protein expressions were abolished by GSK0660 or *L*-NAME (*P*<0.05) (Figs 5 and 6).

### Effect of polydatin on eNOS and iNOS activities in aortas incubated in high glucose

**Fig 7:**
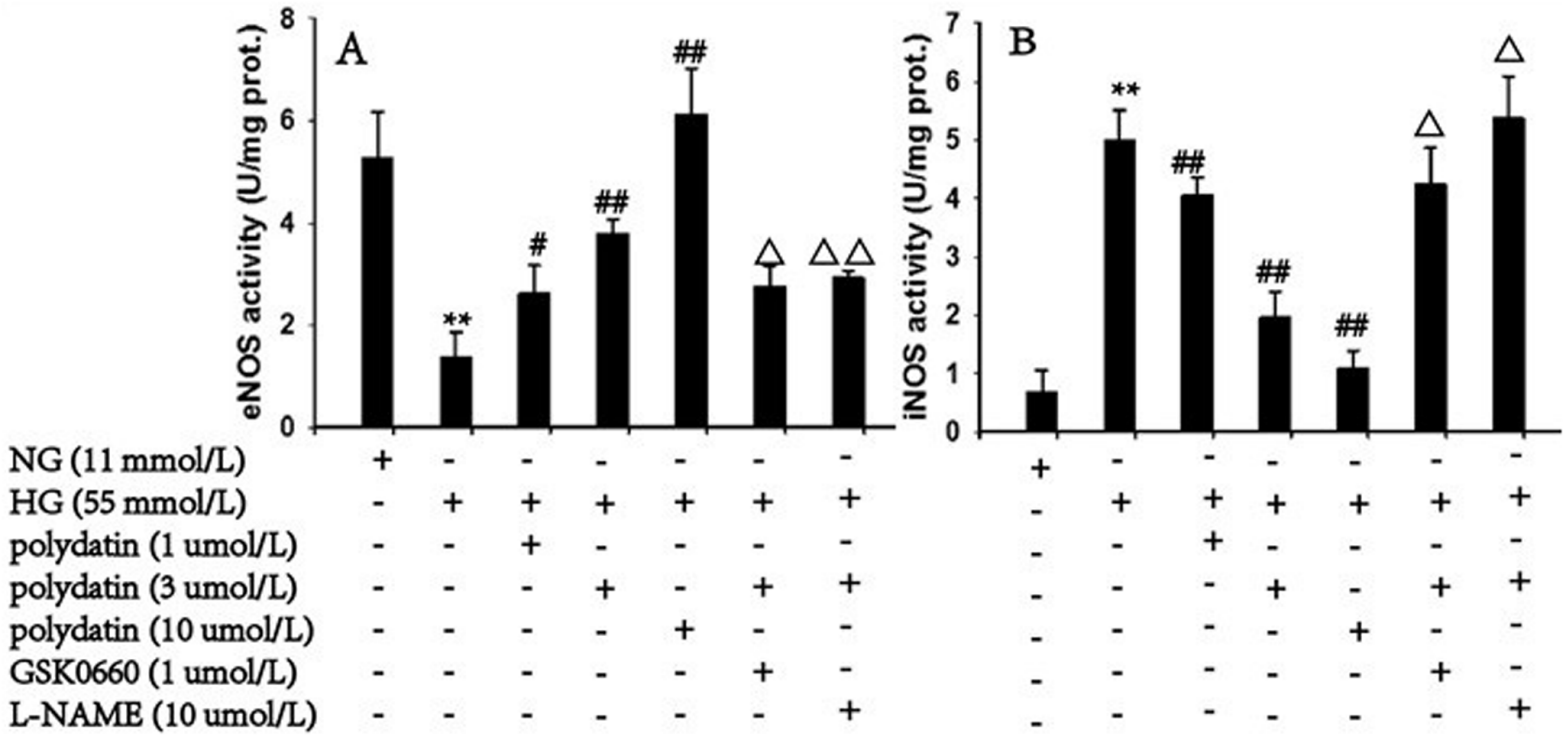
Effect of polydatin on eNOS and iNOS activities in aortas incubated in HG (glucose at 55 mmol/L) conditions. Stimulation by HG caused a significant decrease in eNOS activity (A) and an increase in iNOS activity (B), which were dramatically reversed by polydatin (6, 3, 10 μmol/L) in a concentration-dependent manner. Interestingly, GSK0660 (1 μmol/L) and *L*-NAME (10 μmol/L) could inhibit the effects of polydatin (3 μmol/L). (mean ± S.E.M, n = 6). **P<0.01 *vs*. NG (glucose at 11 mmol/L); ^*#*^ *P*<0.05, ^##^ *P*<0.01 *vs*. HG; ^Δ^ *P*<0.05, ^ΔΔ^ *P*<0.01 *vs* HG+polydatin (3 μmol/L). “+” or “-”: treatment with or without the relevant reagent.

As shown in Fig 7, HG stimulation decreased the eNOS activity by 67.9% and caused a 6.3 fold increase in iNOS activity, which were markedly reversed by treatment with polydatin (1, 3, 10 μmol/L) in a concentration-dependent manner (*P*<0.01). GSK0660 (1 μmol/L) or *L*-NAME (10 μmol/L) blocked the effects of polydatin (3 μmol/L) (*P*<0.05).

### Effect of polydatin on NO and PGI_2_ levels in aortas incubated in high glucose

**Fig 8:**
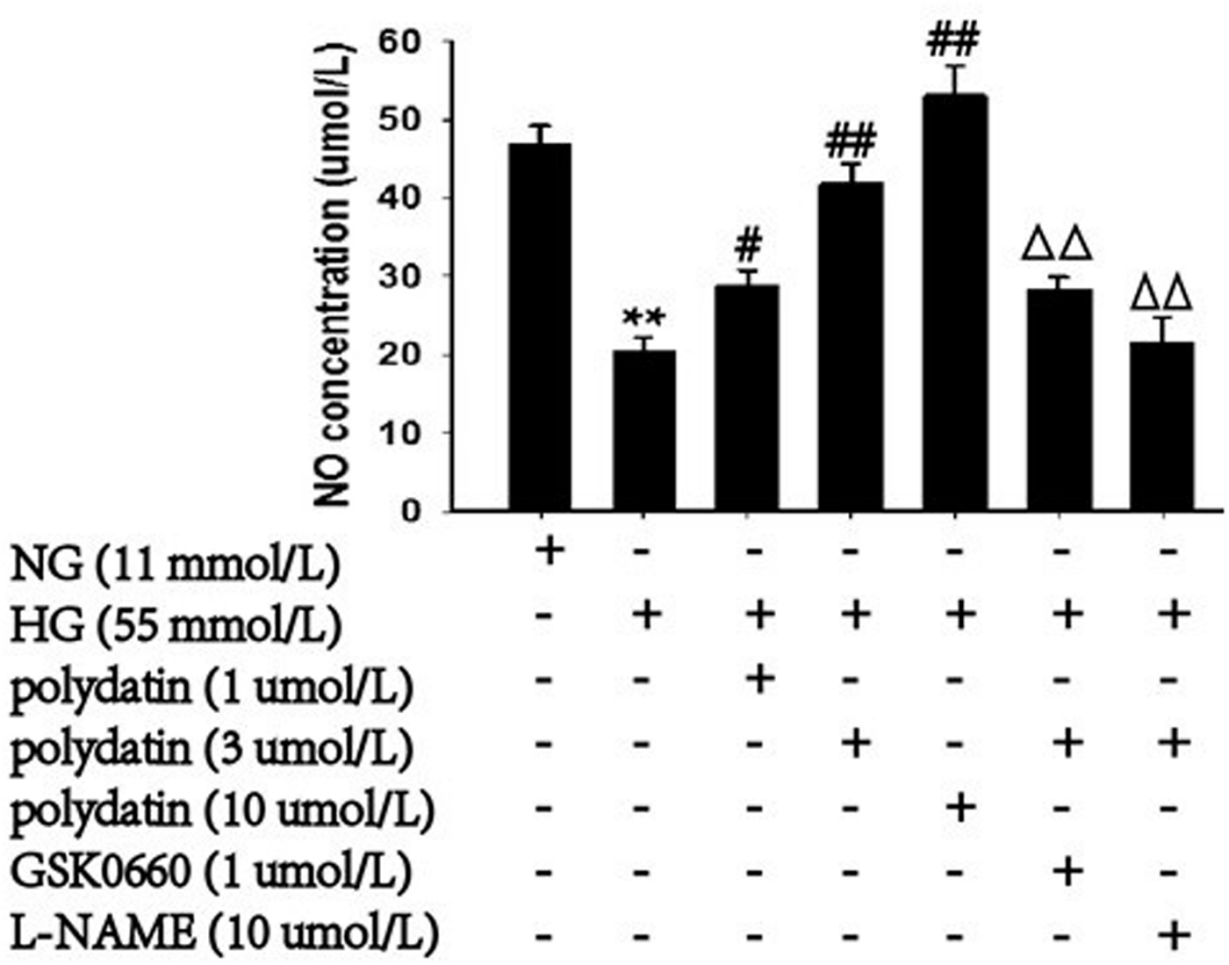
Effect of polydatin on NO level in aortas incubated in HG (glucose at 55 mmol/L) conditions. The decrease of NO in aortas after exposure to HG (glucose at 55 mmol/L) was markedly blocked by polydatin (1, 3, or 10 μmol/L) in a dose-dependent manner. However, the reversion effect of polydatin could be partially inhibited by co-incubation with GSK0660 (1 μmol/L), and completely blocked by *L*-NAME (10 μmol/L). (mean ± S.E.M, n = 6). **P<0.01 *vs*. NG (glucose at 11 mmol/L); *#P*<0.05, ##*P*<0.01 *vs*. HG; ^ΔΔ^ *P*<0.01 *vs*. HG+polydatin (3 μmol/L). “+” or “-”: treatment with or without the relevant reagent.

The NO concentration significantly decreased, by 56.5%, compared with control levels in HG-stimulated aortas (*P*<0.01). This effect was counteracted by polydatin in a concentration-dependent manner (1, 3, 10 μmol/L) (*P* <0.05). However, the PGI_2_ level showed no difference in all groups (data not shown). GSK0660 (1 μmol/L) was able to partially inhibit the improving effects of polydatin (3 μmol/L) on NO synthesis. Notably, *L*-NAME (10 μmol/L) could completely abolish this function of polydatin (*P* <0.01) (Fig 8).

## Discussion

Whereas a clear relationship between DM and microvascular and macrovascular diseases has been well established for decades, the mechanism is incompletely understood. An organism loses the ability to regulate glycometabolism in both types of diabetes, resulting in a sustained hyperglycemic states, which is considered a major risk factor for impairing the endothelium [16–17]. Similarly, our present study indicated that EDR under acetylcholine was damaged by high glucose (55 mmol/L) incubation, which mimicked pronounced hyperglycemia *in vitro*. The alterations were not caused by hyperosmotic conditions, as both the NG and the HG group had the same osmotic pressure. The structural and functional evaluation of endothelial function is based on the normal vasodilative response to acetylcholine; thus, attenuation of the relaxation generated by acetylcholine indicated that the endothelium was damaged, which was verified by examining the aortas’ morphology by HE staining and transmission electron microscope. Unfortunately, there are no treatments to stop this course.

Multiple animal experiments have shown that polydatin has effects on hypertension, ischemia / reperfusion, atherosclerosis, heart failure, diabetes, and aging. In the present study, for the first time, we examined the protective effect of polydatin on EDR under elevated glucose. We showed that the negative effect of high glucose on acetylcholine-induced vasodilation and the destruction of the intima could be restored by polydatin in a dose-dependent manner, indicating that polydatin may be a potent endothelium protective agent for diabetes. How polydatin triggers the signaling cascade to produce this effect is mostly unknown.

In aortas, NO is the most important vasoactive factor accounting for EDR under acetylcholine. The eNOS-NO system is thought to be responsible for endothelial dysfunction in diabetes. In addition, iNOS, another isoform of NOS that is induced by oxidative stress or inflammatory mediators with the strongest NO-generating potency in mammals, contributes to cell injury in multiple diseases. Endothelial iNOS overexpression resulted in severe endothelium injury in transgenic mice. Our results also showed that high glucose incubation led to significant decreases in eNOS expression and NO concentration, with increased iNOS mRNA and protein levels. Previously, polydatin was observed to enhance gene expression and enzyme activity of eNOS, and NO production [18–20]. Consistent with this, our investigation demonstrated that treatment by polydatin could ameliorate the impaired EDR and endothelium by increasing the expression of eNOS, eventually leading to in a beneficial increase in NO production. Meanwhile, polydatin also decreased iNOS expression under high glucose. The beneficial effects of polydatin were blocked completely by *L*-NAME, an eNOS inhibitor. These results suggested that the increase in NO via the eNOS-NO system plays an important role in the protective action of polydatin on damaged EDR in diabetes. PGI_2_ is another important endothelium-derived vasodilator. COX_2_ is the rate-limiting enzyme in PGI_2_ synthesis. However, meloxicam, an inhibitor of COX_2_, had no effect on the function of polydatin. In addition, no changes were observed in any of the groups in response to PGI_2_, which suggested that PGI_2_ is not involved in the damage caused by high glucose or the protective effect of polydatin in this model.

The eNOS-NO system is the target of polydatin in restoring endothelium-dependent acetylcholine-induced relaxation in aortas; however, the upstream factors are unclear. Some observations suggested that an increased in cytosolic Ca^2+^ influx subsequent to disruption of the plasma membrane by high concentrations of resveratrol may result in Ca^2+^-dependent eNOS activation in endothelial F-2 cells, which did not occur in low to moderate concentrations [21]. The glycoside group in the C-3 position of polydatin allows it to more easily enter into cells, where it interacts with multiple targets. PPARβ is one of the three isoforms of PPARs. The highly selective PPARβ agonist GW0742 could restore acetylcholine-induced relaxation in streptozocin-induced type 1 diabetic rats and significantly upregulated both mRNA and protein levels of eNOS and NO production in aortas of diabetic animals [22]. In our investigation, high glucose incubation resulted in significantly lower of PPARβ mRNA and protein expression, which could be reversed by treatment with polydatin in a concentration-dependent manner. Thus, the rescue effects of polydatin on EDR, endothelium morphology, and eNOS, or iNOS expression, and NO production seemed to be related with PPARβ activation to a certain degree because the PPARβ antagonist GSK0660 suppressed them. It could be concluded that the PPARβ-NO signal pathway mediates the effect of polydatin. However, PPARβ may not be the only upstream factor, because GSK0660 could only block the effect of polydatin partially. The functions of other pathways in the effects of polydatin in improving the eNOS-NO system and restoring the impaired EDR induced by high glucose require further study.

In summary, the present study showed that polydatin could improve the impaired EDR induced by high glucose. Mechanistic studies revealed a novel molecular mechanism whereby polydatin acts by promoting the expression of eNOS, enhancing eNOS activity and decreasing the iNOS level, finally resulting in a beneficial increase in the release of NO, which may, at least in part, act through activation of the PPARβ signaling pathway. Our findings should evoke further interest in polydatin as potential therapeutic drug in the prevention of diabetes-related cardiovascular diseases. Nevertheless, the aforementioned effects of polydatin need further verification in diabetes models *in vivo* and in patients.
